# Computational QSAR and structure-based identification of plerixafor-derived PIM-1 kinase inhibitors in diffuse large B-Cell lymphoma

**DOI:** 10.3389/fchem.2026.1798835

**Published:** 2026-06-26

**Authors:** Amritha Thaikkad, B. Angitha, Radul R. Dev, Bristow Ben Joseph, Rajesh Raju, Abhithaj Jayanandan

**Affiliations:** Centre for Integrative Omics Data Science (CIODS), Yenepoya (Deemed to be University), Mangalore, Karnataka, India

**Keywords:** cancer therapeutics, DLBCL, molecular dynamics, PIM-1 kinase, QSAR

## Abstract

**Introduction:**

Diffuse large B-cell lymphoma (DLBCL) is the most common and aggressive subtype of non-Hodgkin lymphoma, with a substantial proportion of patients developing resistance to standard chemotherapy. Chromosomal translocations resulting in overexpression of the serine/threonine kinase Proviral Integration site for Moloney murine leukemia virus (PIM-1) contribute to disease progression, therapeutic resistance, and poor clinical outcomes, establishing PIM-1 as a promising molecular target. Unlike many kinases, the ATP-binding site of PIM-1 lacks a backbone hydrogen bond donor within the hinge region due to the presence of Pro123, conferring unique structural features relevant for selective inhibitor design. Although R-CHOP is the first-line treatment for DLBCL, approximately 30%–40% of patients develop refractory disease, highlighting the need for novel targeted therapies.

**Methods:**

In this study, an in-silico drug repurposing strategy was employed to investigate plerixafor analogues as potential ATP-competitive PIM-1 inhibitors. Quantitative Structure Activity Relationship (QSAR) modeling demonstrated strong predictive performance (R^2^ = 0.82), and molecular docking revealed favorable interactions within the ATP-binding pocket. Molecular dynamics simulations over 200 ns confirmed the dynamic stability of the compound 1–PIM-1 complex, as evidenced by stable Root-Mean Square Deviation (RMSD) values, reduced Root-Mean Square Fluctuation (RMSF) in the active site, and favorable Radius of Gyration (Rg) and Solvent Accessible Surface Area (SASA) profiles.

**Results:**

Additional MM/GBSA, Principal Component Analysis (PCA), Dynamic Cross-Correlation Matrix (DCCM), and free energy landscape (FEL) analyses further supported complex stability and restricted conformational dynamics.

**Discussion:**

Collectively, these findings identify plerixafor-derived compound 1 as a promising PIM-1 inhibitor and provide a robust computational framework for the development of targeted therapeutics against aggressive DLBCL, warranting further experimental validation.

## 1 Introduction

Lymphoma is a heterogeneous group of cancer arising from the clonal growth and proliferation of lymphocytes. This malignancy is classified according to the 2022 World Health Organization (WHO) classification system (5th edition), which integrates morphology, immunophenotypic profiles, and genetic information to delineate distinct, clinically relevant categories (Alaggio et al., 2022). Lymphoma is broadly classified into Hodgkin’s Lymphoma (comprising ≈ 10% of cases) and Non-Hodgkin’s Lymphoma (≈ 90%), encompassing more than 90 subtypes (Mugnaini and Ghosh, 2016), (Paterson and Lambert, 2025). Non-Hodgkin’s Lymphoma is a devastating disease which resulted in 2,60,000 deaths in 2020. The disease usually manifests as painless adenopathy, but systemic symptoms like fever, unexplained weight loss, and night sweats may appear in later or more advanced stages. The most prevalent form of Non-Hodgkin Lymphoma is Diffuse Large B-Cell Lymphoma (DLBCL). Every year, over 1,50,000 new cases of DLBCL are recorded worldwide, accounting for almost 30% of all instances of non-Hodgkin lymphoma in Western nations (D’Alò et al., 2024). DLBCL is a malignant lymphoma that comprises two major subtypes referred to as the Germinal centre B-cell (GCB) subtype and Activated B-cell (ABC) subtype, which are clinically diverse and invasive (Ashpublications, 2016).

The dysregulation of key intracellular signalling pathways often drives the aggressive nature and resistance to treatment in DLBCL. The aggressive clinical behavior and therapeutic resistance observed in Diffuse Large B-Cell Lymphoma (DLBCL) are primarily driven by the dysregulation of key intracellular signaling pathways. Proviral Integration sites for Moloney murine leukemia virus (PIM kinases) are frequently overexpressed in several cancers and are essential in controlling cell death, regulating the growth cycle, and driving proliferation (Rathi et al., 2021). This family comprises three isoforms: PIM-1, PIM-2, and PIM-3. These kinases have different expression patterns in different tissues. The PIM-1 kinases, which belong to the serine/threonine protein kinase family, are currently a common therapeutic target in tumors. They also serve as downstream targets of several signaling pathways, their up and downregulation affects numerous physiological processes. PIM-1 is mostly located in the hematopoietic cells, whereas PIM2 is found in lymphocytes and in the brain. PIM-3 is expressed in kidneys, breast and brain cells (Choudhury et al., 2024; Spitzer and Lazarus, 2025). PIM-1 and PIM-2 share high sequence homology and possess overlapping oncogenic functions (Pierre et al., 2011) while PIM-3 protein shows a high resemblance in the kinase domain along with deficient regulatory domains of the PIM-1 (Barnett et al., 2010). The PIM-1 gene has been found to experience chromosomal translocations that lead to its overexpression specifically in DLBCL, establishing it as a key oncogenic driver.

Structurally, PIM-1 kinase is highly amenable to small molecule inhibition. The N-terminal domain (residues 37-122) is predominantly β-stranded with a single α-helix, while the C-terminal domain (residues 126–305) consists mainly of α-helices, connected by a short hinge region (residues 123-125). The ATP-binding pocket, positioned between the glycine-rich loop (G-loop; residues 44–52), the activation loop (residues 185–204), and the hinge region exhibits a unique structure that it lacks a conventional backbone hydrogen bond donor due to Pro123. Additionally, PIM-1 kinase exists in a constitutively active conformation, independent of phosphorylation. This distinctive feature makes the PIM-1’s ATP-binding site structurally unique and suitable for therapeutic targeting.

Drug repurposing has emerged as a crucial and efficient strategy for identifying novel therapeutic agents. The FDA-approved small molecule inhibitor Plerixafor, a C-X-C chemokine receptor 4 (CXCR4 receptor) antagonist used for haematopoietic stem cell mobilization in NHL and multiple myeloma, represents a promising lead (Brave et al., 2010). This is supported by the crucial molecular connection that PIM-1 functions as a key downstream effector of the CXCR4 signaling pathway mediating cancer survival and contributing to chemoresistance in DLBCL (Sarah et al., 2014). Müller et al. found in their study that PIM-1 inhibition counteracts the compensatory upregulation of the CXCR4 receptor on hematopoietic stem cells (HSCs) following Plerixafor treatment and decreases CXCL12 levels within the bone marrow niche. Combining PIM-1 kinase targeting with CXCR4 inhibition could enhance the collection of stem cells in patients at risk of poor mobilization during treatment (Müller et al., 2016). A key factor in selecting Plerixafor for analogue design is its favorable pharmacokinetic profile and metabolic stability. Also, the lack of metabolism in the liver and its non-influence on cytochrome P450 enzymes.

Leveraging this molecular connection and considering PIM-1’s unique and targetable ATP-binding site, our study proposes to investigate Plerixafor analogues as novel ATP-competitive PIM-1 kinase inhibitors. This study employs an *in silico* drug repurposing approach. The primary objective was to identify analogues with reduced toxicity and enhanced binding affinity compared to the standard inhibitor CX-6258. A multi-step pipeline was implemented, involving Quantitative Structure Activity Relationship (QSAR) and docking analyses to shortlist compounds for Absorption, Distribution, Metabolism, Excretion, and Toxicity (ADMET) evaluation. Following this analysis, molecular dynamics simulations (MDS) were subsequently performed. Post-MDS analyses were then conducted to assess the stability and conformational behavior of the most promising complexes. This integrative approach offers a rational and efficient framework for identifying targeted therapeutic candidates against aggressive DLBCL.

## 2 Methodology

The methodology for the drug repurposing study encompasses various computational methods that are designed to identify and validate potential drug candidates against DLBL by targeting the PIM-1 kinase protein.

### 2.1 Preparation of protein

The crystallographic structure of PIM-1 kinase was retrieved from the Protein Data Bank (PDB ID 5O13) (https://www.rcsb.org/) with a resolution of 2.44 Å (Bogusz et al., 2017). This structure was chosen as it contains the co-crystallized ligand CX-6258, a known small-molecule inhibitor of PIM kinases. Protein preparation was performed in Schrödinger Maestro version 2025-3 using the Protein Preparation Wizard, which involved the addition of hydrogen atoms, removal of water molecules, assignment of zero-order bonds to metals, and formation of disulfide bonds. Further optimization included sampling water orientations and accounting for all possible ligand ionization states. Energy minimization of the entire system was carried out with a convergence threshold of 0.30 Å using the OPLS4 force field (Lu et al., 2021).

### 2.2 Preparation of ligand and grid generation

Ligand preparation was carried out on all 3D structural analogues of plerixafor, which were obtained from the PubChem database (https://pubchem.ncbi.nlm.nih.gov/) using the LigPrep tool. The Epik ionizer was used to create proper protonation states at a pH of 7.0, and the OPLS4 force field was applied to construct tautomerized and desalted ligands (Lalu et al., 2025).

A receptor grid measuring 20 × 20 × 20 Å was generated within the ATP binding site. This grid was positioned in the same position as the ligand coordinates specified in the PDB, corresponding to the placement of the cognate ligand.

### 2.3 Deep Auto QSAR

Auto quantitative structure activity relationship (QSAR) is a machine learning tool in the Schrodinger suite for datasets of several thousand entries, while Deep Auto QSAR is used for larger datasets. Both approaches generate categorical or numerical models using physicochemical and topological descriptors, as well as binary fingerprints (Belghalia et al., 2025; Chouhan and Ajazuddin, 2025; Hazhazi et al., 2019). We generate the model by training on 6,686 PIM-1 kinase inhibitor compounds from ChEMBL (https://www.ebi.ac.uk/chembl/) (Chembl ID-CHEMBL2147) using Schrödinger’s Discovery Informatics and QSAR tools. The ligand dataset was divided into a training set, comprising 75% of the data, and a test set, comprising 25% of the data. The validation of the models was assessed using the root mean square error (RMSE), standard deviation (SD), the accuracy of the training set (*R* ^2^), and the accuracy of the test set (Q^2^) to rank all models. The performance of the model was further analyzed using Mean Absolute Error (MAE) analysis by measuring discrepancies between predicted and observed activities. Model build was used to predict the PIC50 values of Plerixafor analogues.

### 2.4 Virtual screening

Virtual screening of the prepared ligands was performed using the GLIDE module (Bogusz et al., 2017), following a sequential docking protocol, beginning with High-Throughput Virtual Screening (HTVS) which retained 100% of the compounds. The highest-scoring ligands identified during the HTVS were subsequently subjected to Standard Precision (SP) docking, which also retained all ligands. This process was followed by a final refinement utilizing the most accurate Extra Precision (XP) docking and further binding free energy was calculated using MMGBSA in the Prime module. The ligands were ranked based on their highest docking score and binding energy (Shaji et al., 2025; Yang et al., 2021).

### 2.5 ADMET analysis

The ADMET (Absorption, Distribution, Metabolism, Excretion, and Toxicity) profile of the potential drug candidate was predicted using ADMETLab 3.0 (https://admetlab3.scbdd.com) to evaluate its pharmacokinetic and toxicological properties, which are critical for determining its biological efficacy and drug-likeness. Properties of the drug were critically analysed as they directly defined the quality of the compound for further drug development.

### 2.6 Molecular dynamic simulation (MDS)

Molecular dynamic simulation of the apo protein and the ligand bound forms was performed using the Desmond module for a period of 200 ns. The system was built by constructing an orthorhombic water box employing a water molecule modeled using TIP3P as a solvent model; sodium and chloride ions were added to neutralize the system. Simulation was performed at 300 K temperature and 1.01 bar pressure using the Nose-Hoover chain as a thermostat and the Martyna-Tobias-Klein barostat method. The analysis of simulation trajectories carried out by simulation interaction panel where Root-Mean Square Deviation (RMSD), Root-Mean Square Fluctuation (RMSF), Ligand interactions, Solvent Accessible Surface Area (SASA) followed by binding free energy calculation for protein-ligand complexes by MM-GBSA method (Hima et al., 2025).

### 2.7 Principal Component Analysis

One effective method for determining the most important dominant motion patterns within protein-ligand complexes is Principal Component Analysis (PCA) (Zong et al., 2024). For the interpretation PCA relies on two parameters: eigenvectors which define the motion mode and serve as the weighting function for obtaining PCA scores, and eigenvalues, which quantify the associated motion intensity by providing the variance captured by each mode (Saha and Manickavasagan, 2021). Atomic mobility determines the protein’s stability and helps determine protein-ligand complex’s evolution during the simulation is assessed using Principal Component Analysis (PCA) (Giuliani, 2017).

### 2.8 Dynamic cross correlation matrices

The Dynamic Cross-Correlation Matrix (DCCM) provides a crucial insight into the synchronized and mutual fluctuations of protein residues, which is essential for identifying dynamic interaction networks and gaining a deeper understanding of the stability and interaction within the protein-ligand complex (Zong et al., 2024).

### 2.9 Free energy landscape

Free-energy Landscapes (FEL) are essential for analyzing the metastable conformational states of the protein-ligand complex, as they help to map the collection of conformations near the native structure (Papaleo et al., 2009). For sampling the molecular dynamic simulation technique was used. The PC1 and PC2 components of MDS were considered while analysing the conformation dynamics of the protein-ligand complexes. 300 K temperature was set for the calculation and Boltzmann constant was also set for converting the probability density into free energy (kJ/mol). A Free energy calculating formula was used to convert probability density to FEL:

$$G\left(x,y\right)=-R\;\mathrm{Tln}\left(P\left(x,y\right)\right)$$

Where G (x,y) is the free energy of the state at point x and y, kB the Boltzmann constant, K the temperature in kelvin, P (x,y) is the normalized probability density. The joint probability distributions P (x,y) of the systems were obtained by histogramming the trajectory projection onto the PC1 and PC2 coordinates was used to create the two dimensional free-energy landscapes (John et al., 2025).

## 3 Results

A total of 32 analogues of the FDA-approved small molecule plerixafor retrieved from PubChem were subjected to the computational methodology. Subsequent QSAR studies and molecular docking were performed to evaluate their binding affinities, biological activity and potential inhibitory activity against the target protein (Aouidate et al., 2018).

### 3.1 Deep Auto QSAR

The models were developed using data from the CheMBL database to predict pIC50 values, which showed a strong correlation with the experimental results from *in vitro* assays. To develop the model, we used regression mode, facilitating the numerical prediction of the biological activity of novel compounds. The entire dataset was divided into a 75% training set and a 25% test set. A total of 43 models were generated using four algorithms: DenseRegressor (9 models), RandomForestRegressor (10 models), TorchGraphConv (13 models), and XGBoost (11 models). The DeepAuto QSAR ensemble achieved an *R* ^2^ of 0.82 and an RMSE of 0.57.

**FIGURE 1:**
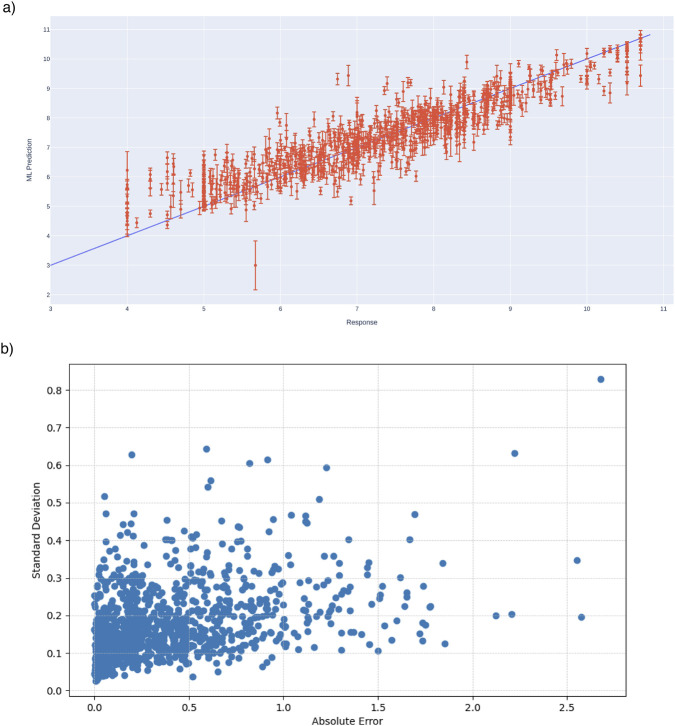
The figure illustrates the performance evaluation of the QSAR model **(a)** The ML prediction vs. response plot depicts a strong linear correlation between the observed and predicted PIC_50_ values. The data points are closely aligned along the regression line, confirming the model’s predictive strength. **(b)** The plot shows the relationship between the absolute error and standard deviation of predicted activities. Majority of compounds cluster within the lower error region, indicating that most predictions fall within the reliable domain of the model.

Figure 1a shows a comparison between the experimental response values (X-axis) and the corresponding ML predicted values (Y-axis) for the dataset. The majority of data points are closely aligned around the diagonal regression line, indicating a strong linear correlation between predicted and observed responses. Only a limited number of outliers deviate, reflecting compounds with unique structural features in the training set. Overall, the narrow spread of most points suggests good predictive accuracy and generalizability on unseen molecules.

To assess the reliability of these predictions, the error versus standard deviation plot (Figure 1b) was examined. This scatter distribution reveals that the majority of molecules exhibit low absolute error (0.0–0.5) alongside low-to-moderate prediction uncertainty, suggesting that the model is not only accurate but also confident in most of its outputs. Compounds with larger errors tend to show higher standard deviation values, indicating that the uncertainty estimates are appropriately calibrated: as the model’s confidence decreases, the error tends to increase. Only a small number of molecules display high error (above 1.5), identifying them as potential outliers with reduced confidence. Overall, this plot indicates robust model performance. Supplementary Table S1 shows the predicted PIC50 values of the Plerixafor analogues generated by our model.

### 3.2 Virtual screening

Virtual screening analyzes the interactions of various ligand conformations with the active site of the protein, subsequently ranking the ligands according to their docking scores and binding energies (Angelova et al., 2025; Li et al., 2025; Walhekar et al., 2026). We performed virtual screening targeting the ATP-binding site of PIM-1 kinase to identify potential lead candidates for DLBCL therapy. The top-ranking analogues, from Table 1, containing the docking and binding energies along with their corresponding interacting residues, prioritized based on docking scores and binding energies, were subsequently validated through MDS. Supplementary Figure S1 represents the 2D chemical structure of the topscoring ligands.

**TABLE 1:** The docking score and the binding energy of the analogues of plerixafor.

Compound	Pubchem ID	Compound name	Docking score (kcal/mol)	Ligand efficiency (LE) (kcal/mol)	MMGBSA (kcal/mol)	Interacting residues
Standard	44545852	CX-6258	−8.73	−0.26	−69.16	Asp128, Lys67
Compound 1	12403258	N,N-bis(3-(N,N-bis(3-aminopropyl)amino)propyl)benzylamine	−13.97	−0.43	−70.38	Asp131, Asn172, Glu171, Val126, Asp186, Asp167, Glu171, Asp131, Asp128
Compound 2	163915843	N'-[2-[[4-[[2-[2-[2-(4-prop-2-enylphenyl)ethylamino]ethylamino]ethylamino]methyl]phenyl]methylamino]ethyl]ethane-1,2-diamine	−13.17	−0.39	−67.29	Gly188, Asp186, Glu171, Asp167, Asp186, and Asp76
Compound 3	10494544	1-[(4-Ethenylphenyl)methyl]-5-methyl-1,5,9-triazacyclododecane	−10.75	−0.48	−61.35	Asp128, Asp131 Glu171, Asp128, Asp131
Compound 4	19775743	N'-(2-aminoethyl)-N'-[2-[2-[(4-phenylphenyl)methylamino]ethylamino]ethyl]ethane-1,2-diamine	−10.71	−0.41	−67.68	Glu171, Asp128, Asp131 Asp128, Asp131
Compound 5	123511629	N'-benzyl-N-[2-[3-(2-methylhepta-2,4,6-trienylamino)propylamino]ethyl]propane-1,3-diamine	−10.70	−0.39	−65.94	Glu171, Asp128, Asp167, Asp186, Asp167, Asp186
Compound 6	138740161	N'-[2-[2-(dibenzylamino)ethylamino]ethyl]ethane-1,2-diamine	−10.62	−0.44	−61.34	Asp128, Asp131, Asp128, Glu171

**FIGURE 2:**
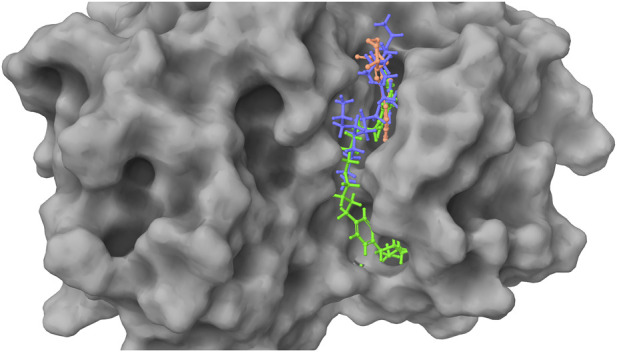
Surface representation of PIM-1 kinase showing the ATP-binding pocket occupied by CX-6258 (orange), compound 1 (violet), and compound 2 (green). The surface view highlights the spatial arrangement of each ligand within the ATP-binding site, enabling direct comparison of their binding orientations and pocket coverage.

CX-6258 shows a docking score of −8.73 kcal/mol and a high binding free energy of −69.16 kcal/mol (Figure 2). It formed 2 H bonds with residues Asp128 and Lys67 (Table 1). Compound 1 (predicted PIC50 = 6.98) exhibited the most favorable binding profile, achieving a docking score of −13.97 kcal/mol and a binding energy of −70.38 kcal/mol. This compound forms 4 hydrogen bonds with Asp131, Asn172, Glu171, and Val126, along with 6 salt bridges involving Asp186, Asp167, Glu171, Asp131, and Asp128. The replacement of the terminal ring with an NH_3_^+^ group from the parent molecule enables an additional salt bridge with Asp128 and allowing the amine to establish a hydrogen bond with Gln127. Compound 2 (predicted PIC50 = 7.07), attained a docking score of −13.17 kcal/mol and a binding energy of −67.29 kcal/mol. It formed 2 hydrogen bonds with Gly188 and Asp186, alongside 4 salt bridges involving Glu171, Asp167, Asp186, and Asp76. The polyamine chain in the derivative, which is absent in the parent compound, played a crucial role in enabling all these residue interactions. Compound 3 (predicted PIC50 = 6.79) demonstrated a docking score of −10.75 kcal/mol and a binding energy of −61.35 kcal/mol, establishing 2 hydrogen bonds with Asp128 and Asp131, as well as salt bridges with Glu171, Asp128, and Asp131. The newly modified NH2 group in the derivative facilitates the interactions with ASP128 and ASP131.

Compound 4 (predicted PIC50 = 6.62) yielded a docking score of −10.71 kcal/mol and a binding energy of −67.68 kcal/mol, forming 4 hydrogen bonds with Glu171, Asp128, and Asp131, in addition to establishing salt bridges with Asp128 and Asp131. The protonated amine groups in the derivative enable these interactions with Glu171, Asp128, and Asp131. Compound 5 (predicted PIC50 = 6.05) achieved docking and binding energy scores of −10.70 kcal/mol and −65.94 kcal/mol, respectively, and formed 4 hydrogen bonds with Glu171, Asn172, Asp128, Asp167, and Asp186, along with salt bridges with Asp167 and Asp186. The protonated amine groups (NH2+ and NH3+) in the derivative facilitate the interaction with these residues. Finally, Compound 6 (predicted pIC50 = 7.44) exhibited docking and binding energy values of −10.62 kcal/mol and −61.34 kcal/mol, respectively. The protonated amine groups of the derivative contribute to the formation of two hydrogen bonds with Asp128 and established salt-bridge interactions with Asp131, Asp128, and Glu171 (Supplementary Figure S2).

Salt bridges play an essential role in drug discovery, as they play a crucial role in enhancing PIM-1-analogues interactions. These interactions contribute to a reduction in conformational flexibility within the binding region, thereby increasing its stability. Such stabilization not only promotes the inhibitory efficacy of the small molecule but also strengthens the overall stability of the protein-ligand complex. An increase in the number of salt bridges between the analogues and the protein may contribute to improvements in both inhibition and stability.

Kinases generally transition between active and inactive states, with activation commonly driven by phosphorylation. However, PIM-1 kinase is distinct in that it naturally adopts an active conformation, phosphorylation is not required for its activation but rather contributes to its structural stability. Instead, phosphorylation serves to stabilize the protein. Studies have shown that PIM-1 kinase residues Lys67, Glu89, Lys169, Asn172, and Asp186 bind with ATP, while Asp167 functions as the catalytic residue for the phosphate transfer from ATP. Interaction of the analogues with Asp167 of the PIM-1 can disrupt ATP binding and thereby inhibit the phosphorylation of residues (Qian et al., 2005).

**FIGURE 3:**
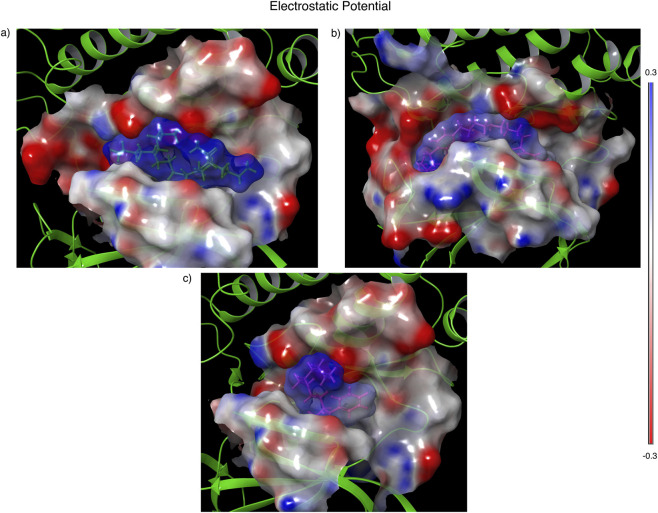
Electrostatic potential of the active site of the PIM-1-ligand complex **(a)** Compound 1 **(b)** Compound 2 **(c)** Compound 6. The red colour shows negative potential and the blue colour shows more positive electrostatic potential.

Within the ATP-binding pocket, acidic residues such as Asp128, Asp131, Asp170, Glu171, Asp234, and Asp239 play a crucial role in ligand stabilization through electrostatic interactions with positively charged compounds. The active site of PIM-1 exhibits a predominantly negative electrostatic potential, which facilitates the attraction of positively charged compounds. Compound 1 shows a greater positive charge, indicated by the blue color, and fits perfectly in comparison to Compounds 2 and 6. This characteristic may have contributed to enhancing binding affinity to the active site (Figure 3). These interactions effectively block ATP binding, thereby inhibiting phosphorylation and downstream signaling. The predicted pIC_50_ values clarify the inhibitory potential of the analogues against PIM-1 kinase. Among them, compounds 1, 2, and 6 exhibit strong docking scores, favorable binding energies, and high pIC_50_ values, strengthening the biological relevance of their docking results. Following this, their ADMET profiles of the analogues were assessed.

The efficiency of ligand docking is employed to optimize and compare compounds of varying molecular sizes (Lang et al., 2025). The standard inhibitor has a more positive value than compounds 1, 2, and 6, likely due to its lower heavy atom count. The binding free energy prediction, used for validating ligand binding to the same binding site, indicates that compound 1 binds more tightly than the standard inhibitor.

### 3.3 ADMET

The ADMET profiles of compounds 1, 2 and 6 were evaluated prior to MD to ensure the selection of candidates with no predicted carcinogenicity and favorable toxicity and absorption characteristics as shown in Table 2. The drawback of most of the drugs against DLBCL is their drug toxicity (Alsubaie et al., 2021; An et al., 2026). Plerixafor also failed to show metabolism in the liver. So we aimed to find an analogue with good ADMET profiles. All compounds followed Lipinski Rule, which is the primary character for a chemical to be considered for drug discovery (Table 2).

**TABLE 2:** The ADMET profile of the top scoring analogues of plerixafor.

ADMET parameters	Compound 1	Compound 2	Compound 6	Recommended range
Molecular weight	449.42	452.36	326.25	100-600
logS	−3.059	−0.977	−1.703	> −4
Lipinski rule	Accepted	Accepted	Accepted	​
Caco-2 permeability	−5.497	−5.683	−5.622	>-5.15
Pgp-inhibitor	---	++	+	0: Non-Inhibitor 1: Non-Substrate
Pgp-substrate	+++	+++	++	0: Non-Inhibitor 1: Non-Substrate
HIA	0.212	+++	+	> 30%
PPB	27.20%	65.20%	73.70%	> 30%
VDss	0.235	6.545	6.351	0.04-20 L/kg
BBB	0.007	–	---	No: Non permeable
CLplasm	6.411	6.395	6.23	>15 ml/min/kg: high clearance < 5 ml/min/kg: low clearance.
T1/2	0.9	0.36	0.284	< 1 h; short Half-life < 1 h; short half-life
hERG Blockers	1	0.9	0.948	0.0
Hematotoxicity	0.024	0.048	0.137	0.0
Carcinogenicity	0.07	0.03	0.059	0.0
DILI	0	0.42	0.077	0: no risk 1: High risk
Human hepatotoxicity	0.758	0.983	0.63	0: Negative 1: Positive

The prediction probability values: 0-0.1 (---), 0.1-0.3 (--), 0.3-0.5 (-), 0.5-0.7 (+), 0.7-0.9 (++), and 0.9-1.0 (+++).

Compound 1 exhibits favorable ADMET profiles, characterized by a molecular weight of 449.42Da. It demonstrates moderate Caco-2 permeability with a value of −5.497, facilitating controlled absorption, and shows no inhibition of P-glycoprotein (0), thereby minimizing the potential for drug interactions. The compound displays low plasma protein binding (27.229%), allowing for elevated free drug concentrations. It also possesses a confined volume of distribution (0.235 L/kg), which is conducive to targeted efficacy, and shows minimal penetration across the blood-brain barrier (0.007), thus reducing the likelihood of central nervous system side effects. With a plasma clearance rate of 6.411 mL/min and a half-life of 0.905 h, the compound provides predictable elimination profiles. Additionally, its toxicity metrics indicate very low hematotoxicity (0.024), carcinogenicity (0.07), and the absence of drug-induced liver injury (0), highlighting its safety potential, even as there remain opportunities for optimization. Among the 3 compounds, compound 1 demonstrated the best scores for all ADMET profiles, indicating its superior safety and minimal toxicity.

### 3.4 Molecular dynamics

MDS are utilized to explore the behavior of protein-ligand complexes within a biological context. In this study, we focused on evaluating analogues of the small molecule plerixafor, which exhibits notable inhibitory activity against PIM-1 kinase (PIC50), along with favorable docking and binding energy characteristics and encouraging ADMET properties. MDS was conducted for a duration of 200 ns to thoroughly evaluate these characteristics.

#### 3.4.1 Root mean square deviation

Root mean square deviation (RMSD) is used to quantify the average spatial displacement of atoms, serving as an important metric for assessing the equilibrium and stability of docked ligands (Gangadharan et al., 2024). It quantifies the average atomic distance between the protein conformations, providing insights into the conformational changes and dynamic behavior (Aier et al., 2016). This parameter quantifies the differences between the Cα coordinates of the protein in its initial and final conformations. A larger RMSD value suggests greater conformational instability, whereas a smaller value indicates enhanced stability. To assess the stability of the protein, a 200 ns molecular dynamics (MD) simulation was conducted (Henna et al., 2025).

**FIGURE 4:**
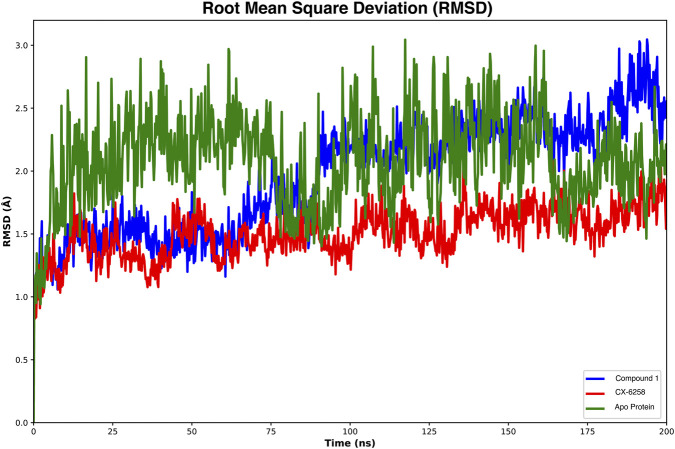
RMSD plot of the Cα over simulation periods of 200ns of the apo protein, and protein complexed with CX-6258 and compound 1.

The apo protein exhibits an average RMSD of 2.08 ± 0.35 kcal/mol, while the CX-6258 and compound 1 demonstrate lower average RMSD of 1.50 ± 0.19 Å and 2.67 ± 0.34 Å, respectively. Initially, the apo protein shows significant fluctuations; however, it stabilizes after 10 ns, maintaining a steady RMSD without fluctuations. Notably, after 75 ns, the RMSD decreases, sustaining the reduced value up to 100 ns. Following this, there is an increase in RMSD, which remains stable until the 160 ns, during which fluctuations are observed yet overall stability is retained. Beyond 160 ns, the RMSD value reduces, averaging at 2 Å. The CX-6258 exhibited a stable trajectory throughout the simulation period. Between 10 ns and 15 ns, a gradual increase in fluctuations was observed, reaching a maximum deviation of 0.50 Å, after which stability was regained. An additional increase of 0.40 Å in the Cα RMSD was observed during the interval from 40 ns to 50 ns, and overall remained stable with only minor fluctuations throughout the simulation. Compound 1 initially showed a rise in the RMSD value with a fluctuation of 0.4Å. After 90 ns, the RMSD value increases to above 2Å and subsequently maintains a consistent trajectory throughout the duration of the simulation, with only a minor deviation observed towards the end of the simulation period (Figure 4).

The CX-6258 and compound 1 showed a lower average RMSD and reduced fluctuation throughout the simulation period. This observation allows us to conclude that the binding of the ligands to the ATP binding region enhances the stability of the protein by reducing its flexibility.

#### 3.4.2 Root Mean Square Fluctuation

**FIGURE 5:**
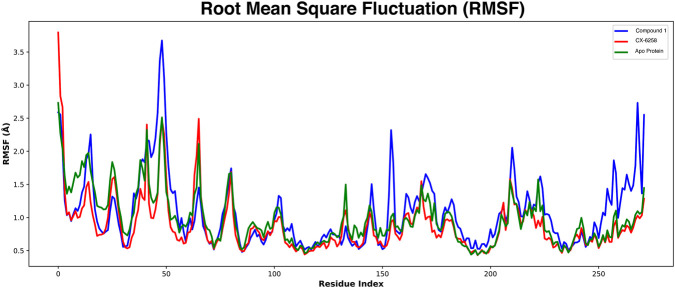
RMSF plot of the Cα over simulation periods of 200ns of the apo protein, and protein complexed with CX-6258 and compound 1.

Root Mean Square Fluctuation (RMSF) is used to understand the atomic displacement of amino acid residues within a protein, providing insights into the dynamic stability of the protein under dynamic environmental conditions. It helps assess the flexibility and conformational heterogeneity of the protein throughout MDS (Ghahremanian et al., 2022). The crystallized structure of PIM-1 kinase begins at residue 34; the software produced the RMSF plot with numbering starting from residue 1. This alignment allowed for a consistent interpretation of domain architecture and facilitated accurate mapping of residue-level fluctuations onto the functional regions of the protein. The protein N-terminal domain (residues 1–86), C-terminal domain (residues 90-269), hinge region (residues 87-89), allowed for a consistent interpretation of domain architecture and facilitated accurate mapping of residue-level fluctuations onto the functional regions of the protein (Figure 5).

The RMSF analysis of both the CX-6258 and compound 1 demonstrates a noticeable similarity to that of the apo protein. RMSF is the measurement of the mobility of individual residues within a molecular structure. The ATP binding region (ligand binding regions) for both compound 1 and the apo protein displayed significantly lower RMSF values. This observation highlights that these regions maintain their rigidity and reduced flexibility, even after ligand binding. Lower RMSF values indicate that the mobility of the residues in the ATP binding region is restricted due to the interaction with the CX-6258 and the compound 1. A modest increase in peak values is observed at residue 50 and in the region beyond residue 250. The latter residues correspond to the α-helical segment of the protein, which is generally considered a more rigid structural component (Bogusz et al., 2017). The fluctuation in this region represents the protein becoming more flexible after the binding of the compound 1.

#### 3.4.3 Ligand interaction

**FIGURE 6:**
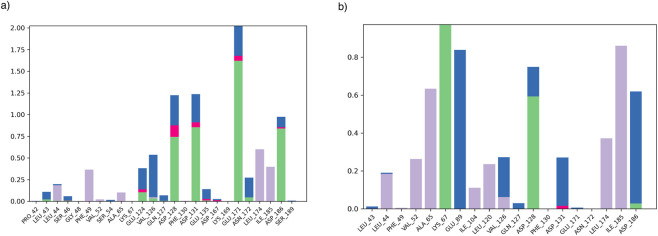
Ligand Interactions of PIM-1 kinase complexed with **(a)** compound 1 **(b)** CX-6258.

The formation of hydrogen bonds is a significant factor influencing the interactions between ligands and proteins, as the stability of the ligand-protein complex is enhanced by an increase in the number of hydrogen bonds. Compound 1, positioned within the ATP binding region, establishes hydrogen bond interactions with the residues Asp128, Asp131, Glu171, and Asp186. These interactions, formed during molecular docking studies, have persisted during molecular dynamics studies. The calculated interaction fraction for all these residues exceeds one, indicating that the ligand maintains these interactions throughout the simulation period (Figure 6). In contrast, the CX-6258 forms a hydrogen bond with Asp128 and Asp186. The interaction within the ATP binding region inhibits the transfer of phosphate from ATP (phosphorylation) and consequently obstructs further downstream processes.

Most protein kinases interact with the ATP binding region by forming two hydrogen bonds. PIM-1 kinase, however, lacks one hydrogen bond donor and instead possesses Pro123 at this position. This distinctive feature renders PIM-1 kinase a promising target for drug design. Notably, only compound 1 forms a hydrophobic bond with Pro123, unlike the inhibitor, which renders the region more rigid compared to the CX-6258, thereby facilitating drug design efforts.

#### 3.4.4 Solvent Accessible Surface Area

**FIGURE 7:**
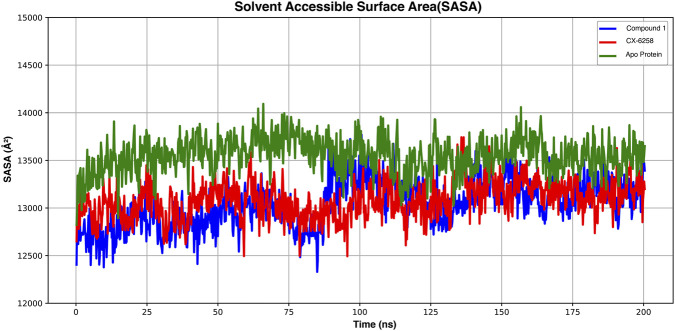
Graph plotting the Solvent Accessibility Surface Area (SASA) of apo protein, CX-6258 and compound 1.

The Solvent Accessible Surface Area (SASA) is a parameter in the prediction of protein folding and stability. It quantifies the surface area that is accessible for ligand binding. SASA enhances the exposure of the contact surface to water molecules, facilitating van der Waals interactions between the ligand and the protein (Ghahremanian et al., 2022). As the value of SASA increases, there is a corresponding increase in the area exposed to water molecules within the biological system, which leads to a decrease in the surface area available for ligand interactions. Thus, an increase in exposure to aqueous environments results in a reduction of the area available for ligand attachment (John et al., 2025; Thaikkad et al., 2025) Average SASA of the apo protein is 13,552.34 Å^2^, whereas the average SASA for the CX-6258 and compound 1 are 13,085.57 Å^2^ and 13,137.78 Å^2^, respectively (Figure 7). This suggests that the decrease in the values for compound 1 and CX-6258 could be due to the presence of the ligand, the surface area exposed to the water will be less compared to the apo protein.

#### 3.4.5 MMGBSA

Utilizing the trajectory generated after MDS, the binding free energy of the complex was assessed using the Molecular Mechanics/Generalized Born Surface Area (MM/GBSA) method. This analysis provides the energy profiles of the formed complex (Table 3). The MM/GBSA was calculated using 10 frames with a step size of 20 ns with starting frame 0 and the ending frame 1001. Compound 1 exhibited an average MM/GBSA value of −69.96 ± 4.40 kcal/mol, while the CX-6258 demonstrated an average of −71.88 ± 5.75 kcal/mol. Even though the CX-6258 exhibits a slightly more negative average value than compound 1, this difference is considered negligible. The MMGBSA value for compound 1 remained constant before and after the MDS, whereas the value for CX-6258 demonstrated a slight increase following the MD simulation. However, due to these minimal variations, both compounds can be affirmed to possess high binding free energy (Table 3).

**TABLE 3:** MMGBSA value and their contributing energies of compound 1 and CX-6258.

Compounds	Bind energy (kcal/mol)	Coulomb energy (kcal/mol)	Covalent energy (kcal/mol)	Hbond energy (kcal/mol)	Lipo energy (kcal/mol)	vdW energy (kcal/mol)
Compound 1	−69.96	−478.49	4.77	−4.29	−18.39	−41.46
CX-6258	−71.88	−64.00	0.84	−0.95	−27.69	−50.82

Hbond- Hydrogen bond, Lipo- Lipophilic energy, vdW- van der waals energy.

The highest energy contribution is by Coulombic energy in the energy profile of both compound 1 (−478.49 kcal/mol) and the CX-6258 (−64 kcal/mol). The more negative coulombic energy associated with compound 1 serves as an indicator of the attractive electrostatic energy, increasing the binding potential of the analogue with the protein.

#### 3.4.6 Dynamic Cross-Correlation matrix

**FIGURE 8:**
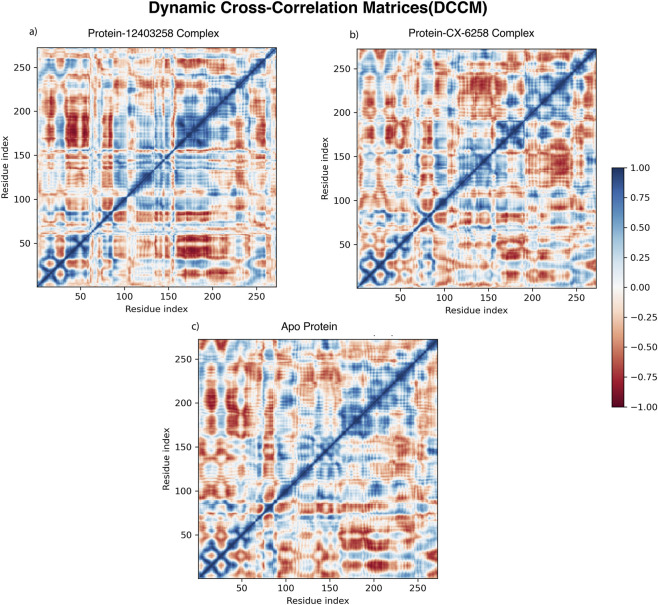
Dynamic Cross-Correlation matrices of **(a)** compound 1, **(b)** CX-6258, **(c)** apo protein.

The Dynamic Cross-Correlation Matrix (DCCM) was used to examine the coordinated motions of protein residues over the 200 ns simulation. In the plot, blue regions represent positively correlated motions, where atoms move together in the same direction, while red regions indicate negative correlations, reflecting movement in opposite directions (Rana et al., 2025). For Compound 1, a pronounced positive correlation is observed between residues 110–150 and 160–225, the ATP-binding region (Figure 8). This correlation pattern is notably stronger than that seen for CX-6258 and the apo protein (Zhang et al., 2023). Overall, the DCCM analysis suggests that Compound 1 enhances stability within this region through these strong positive correlations.

#### 3.4.7 Principal Component Analysis

**FIGURE 9:**
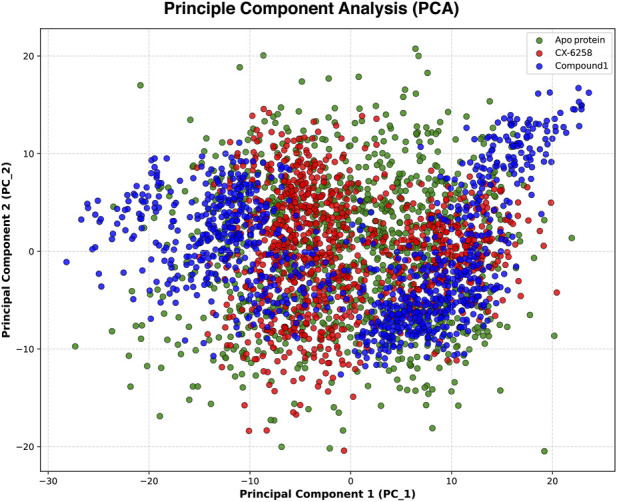
Principal Component Analysis (PCA) of apo protein in green dots, PIM-1 bound CX-6258 in red dots and compound 1 in blue dots.

Principal Component Analysis (PCA) is employed to represent the protein-ligand complex in a significantly simplified form over the simulation period (Kundil et al., 2025). The clusters are useful in understanding the stability and flexibility of proteins. A more compact cluster indicates greater rigidity and reduced flexibility. In this analysis, the apo protein clusters are dispersed over a broad area. In contrast, the clusters associated with the CX-6258 and compound 1 are confined to a smaller area when compared to the apo form. This indicates that the protein attains a more rigid conformation following the binding of the CX-6258 and compound 1 (Figure 9).

#### 3.4.8 Free energy landscape (FEL)

According to the funnel property of proteins within their energy landscape, there exists a single point analogous to the lowest point of a funnel, where proteins achieve stability. The global minimum represents the point of lowest energy within a structure, indicating its most stable configuration at minimal energy. In contrast, local minima refers to points where the energy is lower than that of the surrounding environment. Ben-Naim asserts that the protein–solvent system must conform to this funnel structure. However, Li-Quan and colleagues contend that the ruggedness of a protein is influenced by its energy landscape, which subsequently governs its thermodynamic and kinetic properties (Yang et al., 2013). Thus, a multifunnel landscape reveals additional minima beyond the global minimum, providing insights into the kinetic and thermodynamic behaviors of proteins. This multifunnel configuration embodies multiple functions and possesses the potential to act as molecular switches, thereby stabilizing various motifs (Salhi and Queen, 2004).

**FIGURE 10:**
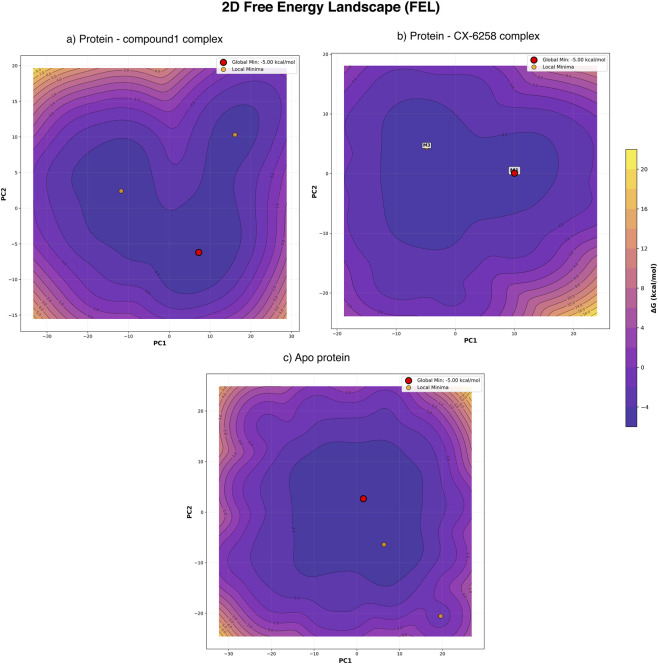
2D Free Energy Landscape of **(a)** protein-compound 1 **(b)** protein-CX-6258 and **(c)** apo protein.

The FEL analysis of the apoprotein reveals a metastable system characterized by the presence of multiple energy minima. Three stable basins were identified, representing conformational states with enhanced stability relative to other confirmations during the MD simulations, notably at frames 555 and 304. In contrast, the FEL of the protein–CX-6258 complex demonstrates a metastable conformational profile with two low-energy minima, corresponding to energetically favorable states observed at frames 808 and 285. Similarly, the FEL associated with compound 1 exhibits three distinct minima, indicating low-energy conformations identified at frames 527, 286, and 899. The protein–compound 1 complex exhibited an additional low-energy conformation compared to the apoprotein and the protein–CX-6258 complex, indicating enhanced conformational stability of the compound 1–bound system relative to the other complexes (Figure 10).

**FIGURE 11:**
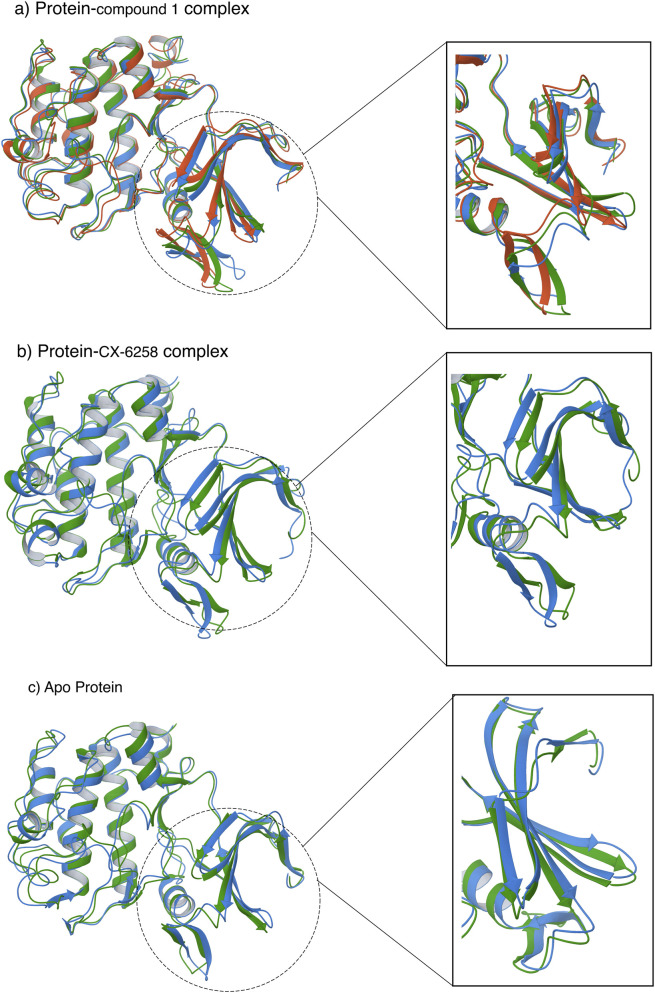
Superimposed structures extracted from the minimum energy frames of the MD trajectories for the PIM-1 complex with **(a)** compound 1, **(b)** the CX-6258, and **(c)** the apo protein.

Figure 11 shows images of the protein confirmation corresponding to the minima frames, with the fluctuating region depicted in the box. The green colour is confirmation in the frame of the highest minima and the blue and brown are the colour of the other confirmations in the frames of the minimas, by overlapping these frames, small fluctuations are only seen in the beta sheets of the N terminal which may be due to the fluctuations in the terminal, which may responsible for the slight differences in free energy.

## 4 Discussion

DLBCL is an aggressive and rapidly proliferating subtype of lymphoma, classified as medium to high grade. Despite its aggressive treatment DLBCL possesses a substantial clinical challenge due to inherent resistance and high relapse rate (Wang et al., 2023). Although the standard care for DLBCL relies on conventional chemotherapeutic regimens, these are ineffective in nearly 30%–40% of patients, resulting in high disease-associated mortality. Research has shown that the overexpression of PIM-1 plays a critical role in DLBCL by driving malignant cell proliferation through dysregulation of the cell cycle and inhibition of apoptosis, thereby making it a promising therapeutic target. Importantly, PIM-1 functions not only as a kinase but also as a transcription factor and an activator of other transcription factors, including signal transducer and activator of transcription 3 (STAT3), which further enhances PIM-1 gene expression (Bachmann and Möröy, 2005). PIM-1 promotes cancer cell survival by phosphorylating the endogenous apoptosis signaling kinase (ASK1), which prevents the activation of pro-caspase-3. However reduced PIM-1 expression decreases the phosphorylation of BAD, leading to caspase-3 cleavage and the induction of apoptosis (Gu et al., 2009).

In this study, we aimed to identify analogues of plerixafor capable of inhibiting PIM-1 kinase by blocking ATP binding and thereby preventing phosphorylation of residues involved in downstream signaling. Docking results demonstrated that plerixafor analogues exhibited strong binding affinity within the ATP-binding pocket of PIM-1. Compounds 1 and 2 showed the highest docking and binding energy among the analogues. Furthermore, *in silico* ADMET analysis of these compounds provided valuable insights into their drug-likeness profiles. While compounds satisfied Lipinski’s criteria, potential challenges were flagged, including predicted hERG inhibition and hepatotoxicity, which remain major liabilities in kinase drug development. By development. By analysing all these, compound 1 appeared as a potential ligand with good ADMET profiles. The mixed permeability and efflux profiles observed also suggest that optimization may be required to enhance cellular uptake and reduce resistance mediated by P-glycoprotein.

QSAR analysis enhanced the docking and ADMET findings by establishing quantitative relationships between structural descriptors and biological activity. The developed model demonstrated a strong correlation (*R* ^2^ = 0.82). Notably, compounds with higher predicted activity in the QSAR model also showed favorable docking energies and interaction profiles within the ATP-binding pocket, reinforcing the reliability of the computational predictions. This alignment between QSAR-predicted activity (PIC50 = 6.98) and binding energetics strengthens confidence in compound 1 as a potential lead molecule.

MD simulations confirmed that the protein–ligand complexes remained dynamically stable over the 200 ns trajectory, as evidenced by consistent RMSD values, reduced RMSF in the ATP-binding region, and favorable Rg and SASA profiles. These results suggest that ligand binding not only stabilizes the kinase but also promotes a more compact protein conformation, reducing solvent exposure and improving complex rigidity. Compound 1 established multiple hydrogen bonds and salt bridges with key residues, including Asp128, Asp131, Glu171, and Asp186—interactions essential for kinase inhibition. In contrast to the CX-6258, compound 1 also formed a unique hydrophobic interaction with Pro123, a hinge region residue specific to PIM-1 but absent in many other kinases. This interaction may provide a selectivity advantage, thereby enhancing the druggability of PIM-1. Energy decomposition from MM/GBSA further emphasized the predominant contribution of Coulombic interactions to the stabilization of the compound 1 complex, with binding free energies comparable to the CX-6258. Importantly, DCCM and PCA analyses revealed restricted conformational changes and limited protein motion upon ligand binding, consistent with effective inhibition of kinase activity. Additionally, FEL provided insights into energy minima and the stabilization of the protein-ligand complex.

Plerixafor and its analogues present a distinct therapeutic advantage over other FDA-approved treatments for Non-Hodgkin’s lymphoma (NHL) due to their unique mechanism of action. By inhibiting CXCR4, plerixafor analogues disrupt the interactions between tumors and the microenvironment. Moreover, the targeted approach of CXCR4 holds promise in overcoming drug resistance, a significant limitation associated with conventional NHL therapies. Since these analogues function through a signaling pathway rather than directly damaging DNA or microtubules, they possess a different toxicity profile compared to CX-6258 chemotherapy. Lastly, the capacity of plerixafor to synergize with additional targeted or immunotherapeutic agents renders its analogues particularly appealing for combination treatment strategies in diffuse large B-cell lymphoma (DLBCL). However, further *in vitro* biochemical validation, kinase selectivity profiling, and experimental toxicity assays are essential to translate these computational findings into viable therapeutic candidates.

## 5 Conclusion

Computational analyses revealed that plerixafor analogues, particularly compound 1, exhibit strong binding affinity and stable interactions within the ATP-binding site of PIM-1 kinase. MDS confirmed the structural stability and compactness of the complex, while MM/GBSA analysis indicated favorable binding energetics comparable to CX-6258. ADMET evaluation suggested acceptable drug-likeness. Overall, compound 1 represents a promising small-molecule lead for targeted PIM-1 inhibition in DLBCL. Future studies should focus on experimental validation, such as enzyme inhibition assays or biophysical methods to elucidate its binding affinity towards PIM-1 and exploring synergistic potential with existing therapies to advance these small molecules toward clinical application. Further *in vitro* and *invivo* efficacy experiments needed to prove its safety and efficacy.

## Data Availability

The original contributions presented in the study are included in the article/Supplementary Material, further inquiries can be directed to the corresponding authors.
