# Supplementary material for: Computational QSAR and structure-based identification of plerixafor-derived PIM-1 kinase inhibitors in diffuse large B-Cell lymphoma
Source: Front Chem. 2026 Jun 26;14:1798835. doi: 10.3389/fchem.2026.1798835 (PMC13353055; doi:10.3389/fchem.2026.1798835)
Supplement: Supplementary file 1 [file Table1.DOCX]

**Computational QSAR and Structure-Based Identification of Plerixafor-Derived PIM-1 Kinase Inhibitors in Diffuse Large B-Cell Lymphoma**

Amritha Thaikkad^1^, Angitha B^1^, Radul R Dev^1^, Rajesh Raju^1*^, Abhithaj Jayanandan^1*^

^1^Centre for Integrative Omics Data Science (CIODS), Yenepoya (Deemed to be University), Mangalore, Karnataka (575018), India

**Supplementary Table 1** shows the predicted task score (PIC50) of the analogues predicted using the QSAR model

| **Compound ID** | **PredictTask score** |
| --- | --- |
| 138740161 | 7.443 |
| 57578606 | 7.344 |
| 57578639 | 7.308 |
| 91215114 | 7.128 |
| 11646845 | 7.101 |
| 69240417 | 7.098 |
| 163915843 | 7.077 |
| 18175313 | 7.052 |
| 22256402 | 6.991 |
| 12403258 | 6.981 |
| 101475414 | 6.979 |
| 19734928 | 6.971 |
| 15153743 | 6.951 |
| 58260330 | 6.909 |
| 57578619 | 6.854 |
| 115230569 | 6.836 |
| 43252851 | 6.823 |
| 10494544 | 6.792 |
| 144963152 | 6.673 |
| 19775743 | 6.622 |
| 15664417 | 6.615 |
| 10619111 | 6.592 |
| 69239334 | 6.585 |
| 11710661 | 6.35 |
| 142601662 | 6.306 |
| 2056891 | 6.261 |
| 123511629 | 6.052 |
| 2055595 | 5.863 |
