# Supplementary material for: Computational QSAR and structure-based identification of plerixafor-derived PIM-1 kinase inhibitors in diffuse large B-Cell lymphoma
Source: Front Chem. 2026 Jun 26;14:1798835. doi: 10.3389/fchem.2026.1798835 (PMC13353055; doi:10.3389/fchem.2026.1798835)
Supplement: Supplementary file 2 [file DataSheet1.docx]

**Computational QSAR and Structure-Based Identification of Plerixafor-Derived PIM-1 Kinase Inhibitors in Diffuse Large B-Cell Lymphoma**

Amritha Thaikkad^1^, Angitha B^1^, Radul R Dev^1^, Rajesh Raju^1*^, Abhithaj Jayanandan^1*^

^1^Centre for Integrative Omics Data Science (CIODS), Yenepoya (Deemed to be University), Mangalore, Karnataka (575018), India


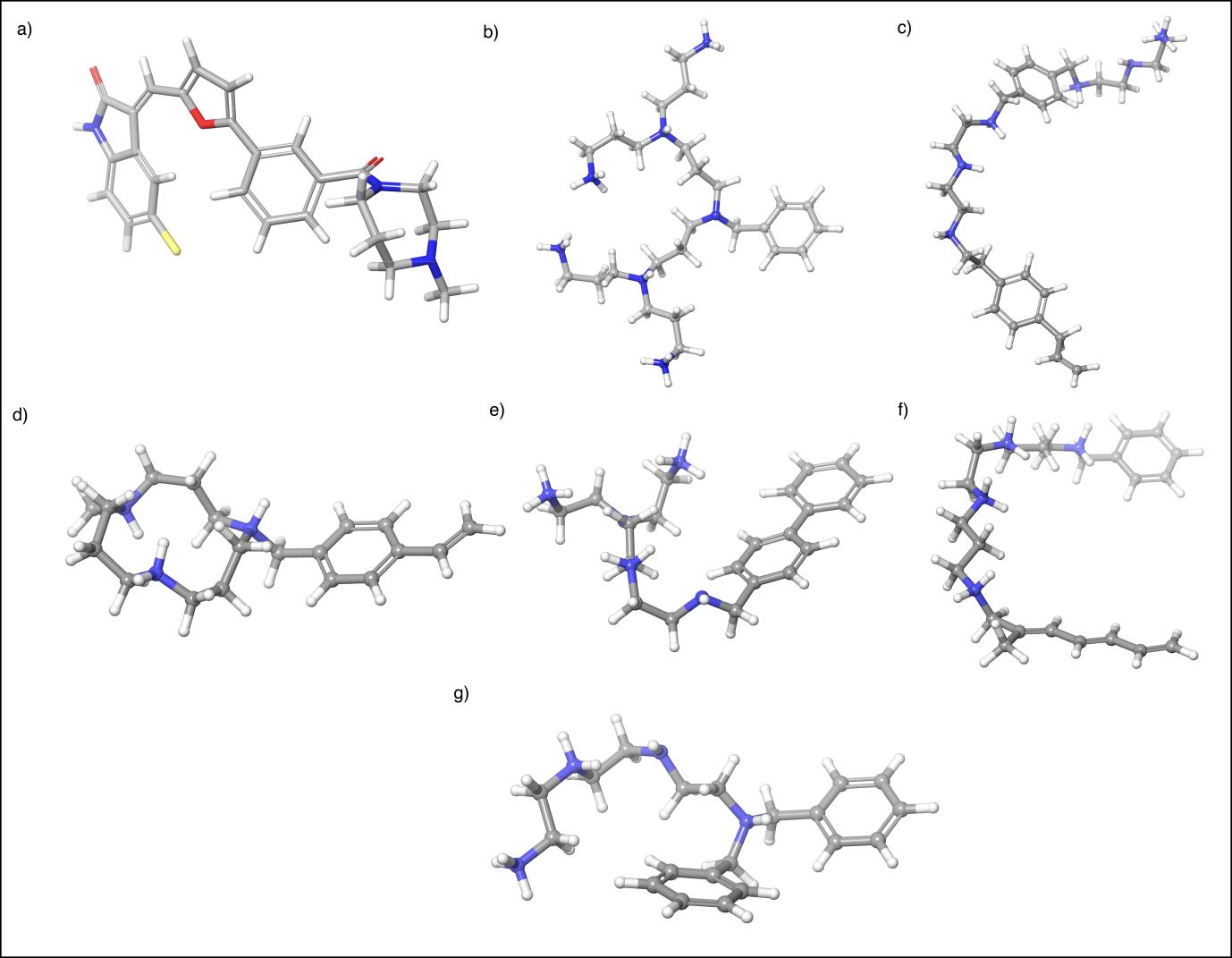


Supplementary Fig.1: 2D chemical structure of a) Standard b) compound 1 c) compound 2 d) compound 3 e) compound 4 f) compound 5 and g) compound 6.


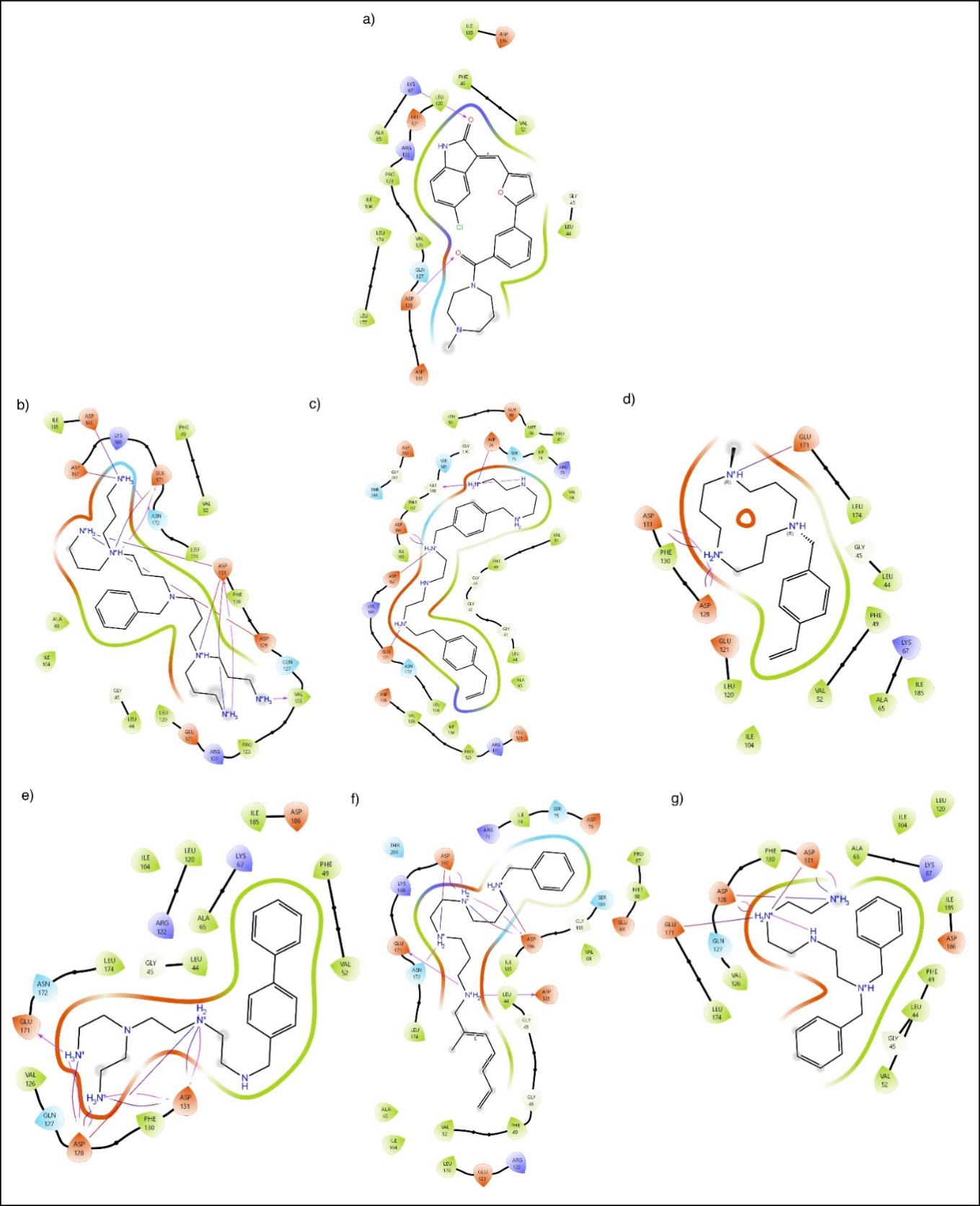


Supplementary Fig.2: Ligand Interaction diagram of a) Standard b) compound 1 c) compound 2 d) compound 3 e) compound 4 f) compound 5 g) compound 6 with PIM-1 kinase.
